# MSHF: A Multi-Source Heterogeneous Fundus (MSHF) Dataset for Image Quality Assessment

**DOI:** 10.1038/s41597-023-02188-x

**Published:** 2023-05-17

**Authors:** Kai Jin, Zhiyuan Gao, Xiaoyu Jiang, Yaqi Wang, Xiaoyu Ma, Yunxiang Li, Juan Ye

**Affiliations:** 1 Eye Center, The Second Affiliated Hospital, School of Medicine, Zhejiang University, Zhejiang Provincial Key Laboratory of Ophthalmology, Zhejiang Provincial Clinical Research Center for Eye Diseases, Zhejiang Provincial Engineering Institute on Eye Diseases, Zhejiang, Hangzhou, 310009 China; 2 College of Control Science and Engineering, Zhejiang University, Hangzhou, 310027 China; 3 College of Media, Communication University of Zhejiang, Hangzhou, 310018 China; 4 Institute of Intelligent Media, Communication University of Zhejiang, Hangzhou, 310018 China; 5 College of Computer Science and Technology, Hangzhou Dianzi University, Hangzhou, 310018 China

**Keywords:** Medical imaging, Eye diseases

## Abstract

Image quality assessment (IQA) is significant for current techniques of image-based computer-aided diagnosis, and fundus imaging is the chief modality for screening and diagnosing ophthalmic diseases. However, most of the existing IQA datasets are single-center datasets, disregarding the type of imaging device, eye condition, and imaging environment. In this paper, we collected a multi-source heterogeneous fundus (MSHF) database. The MSHF dataset consisted of 1302 high-resolution normal and pathologic images from color fundus photography (CFP), images of healthy volunteers taken with a portable camera, and ultrawide-field (UWF) images of diabetic retinopathy patients. Dataset diversity was visualized with a spatial scatter plot. Image quality was determined by three ophthalmologists according to its illumination, clarity, contrast and overall quality. To the best of our knowledge, this is one of the largest fundus IQA datasets and we believe this work will be beneficial to the construction of a standardized medical image database.

## Background & Summary

Fundus photography is the most widely used imaging modality for screening diabetic retinopathy (DR), glaucoma, age-related macular degeneration, and other eye diseases^1^. With the development of artificial intelligence (AI), automatic disease screening via fundus imaging has become a popular topic for researchers and clinical practitioners^2,3^. Many algorithms have been investigated, and some have already been used in clinical practice^4–6^. The quality of fundus images is key to the performance of diagnosis models, as an important preliminary step. Therefore, image quality assessment (IQA) is vital for automated systems.

The most reliable method to assess an image quality requires doctors to assess the original images, but it entails a heavy workload. Over the past years, automated IQA has been developed to score the fundus images^7–10^. Once a model is trained, it can produce fast and real-time predictions, improve the workflow and optimize image acquisition, making the whole process more efficient. To train an excellent model, a well-collected dataset is very important.

Several fundus IQA datasets, which are summarized in Table 1, have been established for public use: DRIMDB^11^, Kaggle DR Image Quality^12^, EyeQ Assessment^13^, DeepDRiD^14^, etc. Many IQA studies have been carried out on these datasets^15^.

**Table 1:** Summarization of publicly available fundus image quality assessment datasets.

Dataset	Year	Number	Quality label	Disease	Annotators
DRIMDB	2014	216 CFP images	‘good’, ‘bad’ or ‘outlier’	DR, non-DR	NA
Kaggle DR Image Quality	2018	88702 CFP images	‘good’ or ‘poor’ for overall quality	DR, non-DR	NA
EyeQ Assessment	2019	28792 CFP images	‘good’, ‘usable’ or ‘reject’ for overall quality	DR, non-DR	2
DeepDRiD	2022	2000 CFP images and 256 UWF images	score criteria 0~10 for artifact, clarity, field definition and 0/1 for overall quality	DR, non-DR	3
MSHF (proposed)	2022	500 CFP images, 500 UWF images and 302 portable camera images	0/1 for illumination, clarity, contrast and overall quality	DR, healthy, glaucoma	3

Nevertheless, these popular datasets have some drawbacks:

- First, the variety of fundus images is limited. In clinical practice, fundus images have various forms, and different kinds of imaging approaches meet the variable needs of clinical practitioners. For example, color fundus photography (CFP) is a widely used kind of fundus image for screening of various ocular disorders. The portable fundus camera is a convenient, hand-held device designed for use in rural areas, and has played an important role in the development of telemedicine. However, the images may lack lesion details, and artifacts are commonly found^16^. Ultrawide-field (UWF) imaging is an advanced fundus photography technique, and is becoming more and more popular in clinical scenarios. UWF machines are always costly, though, and cost-effectiveness remains an important consideration. Therefore, an ideal fundus image dataset should consider the above clinical scenarios.
- Second, the criteria to decide the image quality is not very clear. Most datasets only considered the overall quality, making the label rather subjective and not explainable enough. Liu *et al*.^14^ provided a solution by using a scoring criteria according to artifact, clarity, field definition and overall quality, making the image quality appear more objective. The detailed quality standard makes the label more persuasive.
- Third, existing fundus IQA datasets are based on DR image datasets, and fundus images of other retinal diseases as well as healthy volunteers were not considered. Some fundus diseases may affect the judgment of image quality due to the lesion of the disease. It’s significant for IQA datasets to increase the variety of fundus diseases.

Taking all of these factors into consideration, a public fundus IQA dataset consisting of various forms of fundus images from patients and healthy volunteers with detailed quality labels would be fundamental.

In this paper, we propose a multi-source heterogeneous fundus (MSHF) dataset that contains 500 CFP, 302 portable camera images and 500 UWF images with various source domains from DR and glaucoma patients as well as normal people. For each image, 4 labels are provided: illumination, clarity, contrast and overall quality. Our major contributions can be summarized as follows:

- **Image**: The dataset is composed of sub-databases collected from different devices with diverse appearance patterns, including 500 CFP, 302 portable camera images and 500 UWF images of normal eyes and 2 different eye diseases.
- **Labels**: Each image is labelled by illumination, clarity, contrast and overall quality with 0 or 1, and the dataset has 5208 labels in total.

We believe that the publication of the MSHF dataset will considerably facilitate AI-related fundus IQA research and promote translation from technology to clinical use.

## Methods

**Fig. 1:**
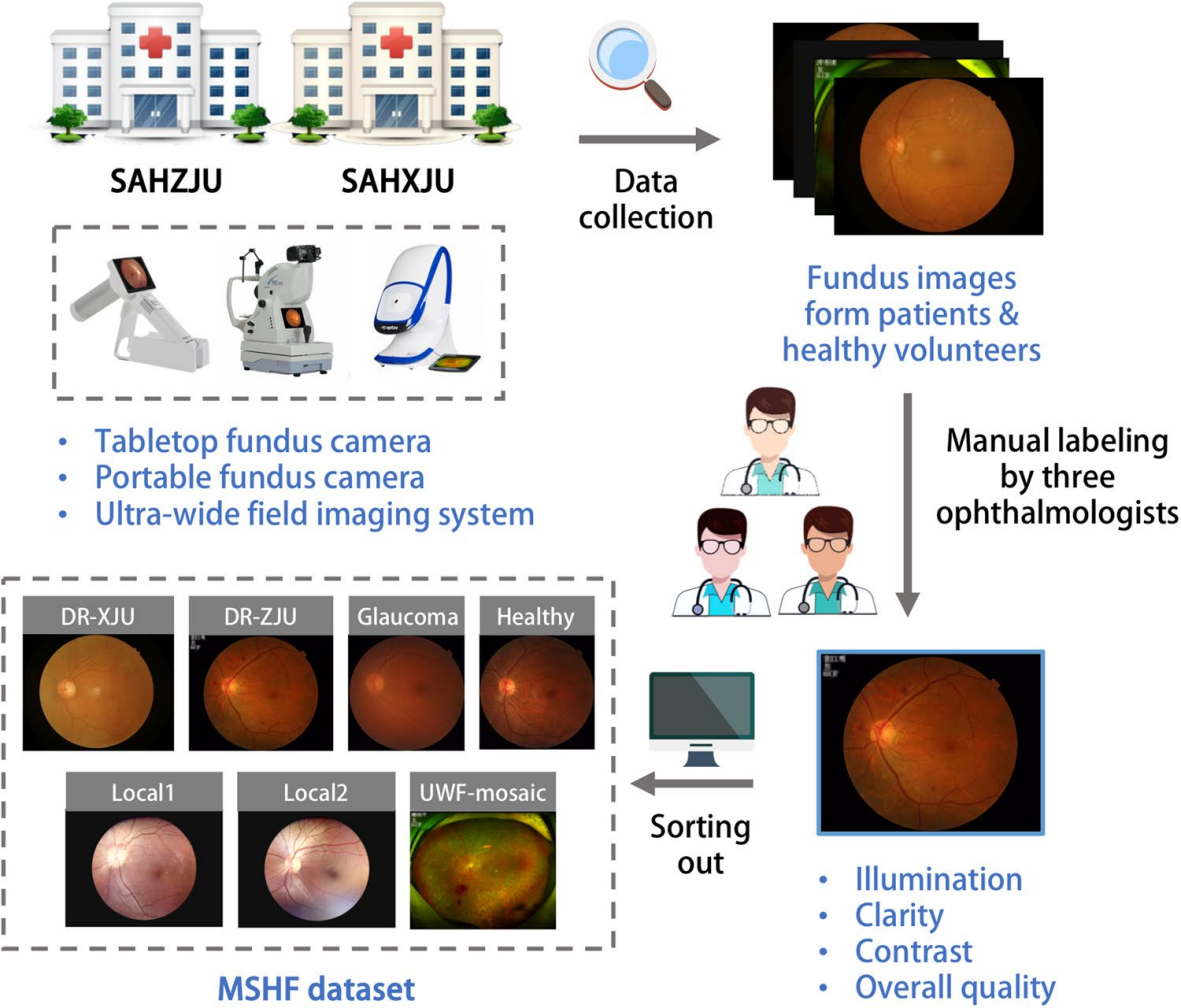
An overview of the study approach and methodology.

An overview of the study approach and methodology is presented in Fig. 1.

### Data collection

A total of 1302 images were retrospectively collected to form 7 sub-datasets: DR-XJU, DR-ZJU, Glaucoma, Healthy, Local1, Local2 and UWF-mosaic. There are three types of images in these datasets: CFP images, portable camera images and UWF images. These images are from 904 patients, with ages ranging from 21 to 77 years. Written consent was signed by every participant before examinations to inform them that the images would be used for research purpose. Ethical approval for the study was obtained from the Ethics Committee of ZJU-2.

Specifically, DR-XJU, DR-ZJU, Glaucoma and Healthy subsets contained CFP images that were centerfield, including the optic disc and the macular area.

Images of DR-XJU were collected from patients with diabetic retinopathy at the Second Affiliated Hospital of Xi’an Jiaotong University (SAHXJU), captured with a Kowa non-mydriatic fundus camera (Kowa Company, Tokyo) with 45 degrees fields of view (FOV) and at 1924 by 1556 pixels.

Images of DR-ZJU, Glaucoma and Healthy were respectively collected from patients with diabetic retinopathy, glaucoma or no disease diagnosed at the Eye Center at the Second Affiliated Hospital of Zhejiang University School of Medicine (SAHZJU). The imaging device was a tabletop TRC-NW8 fundus camera (Top-Con Medical Systems, Tokyo) with 50 degrees FOV and a resolution of 1924 by 1556 pixels.

Local 1 and Local 2 contained portable camera images from healthy volunteers, and the imaging field included centerfield and other locations. These datasets were collected at the Eye Center at SAHZJU, captured with a DEC200 portable fundus camera (Med-imaging Integrated Solution Inc., Taiwan) with 60 degrees FOV and a resolution of 2560 by 1960 pixels. The difference between Local1 and Local2 was the imaging time period.

UWF-mosaic included UWF images from diabetic retinopathy patients. This dataset was also collected at the Eye Center at SAHZJU, and the capture device was an Optos ultra-wide field imaging system (Optos Plc Fife, Scotland) with 200 degrees FOV and a resolution of 1924 by 1556 pixels.

**Fig. 2:**
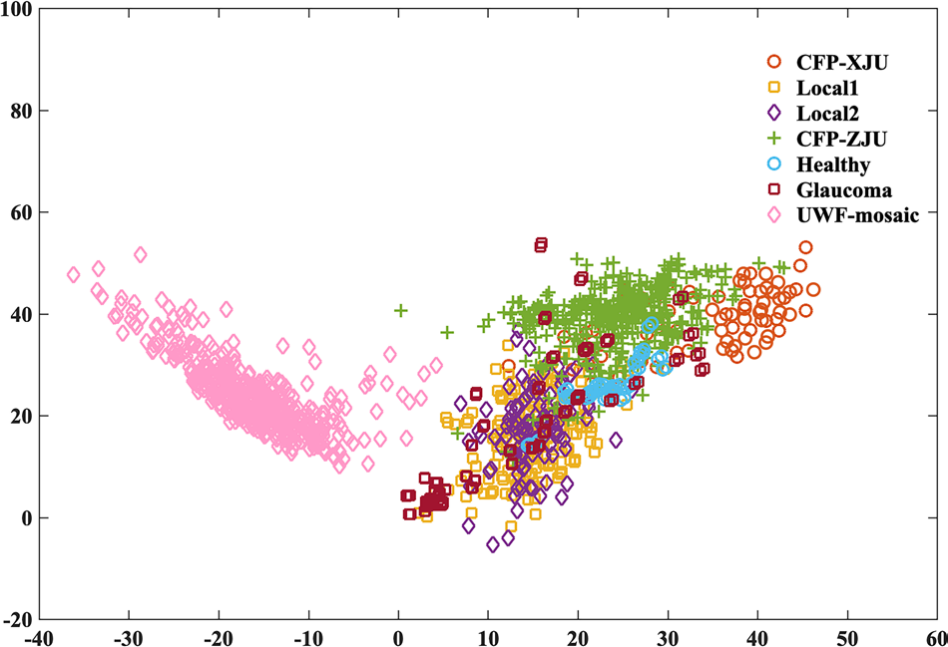
Spatial scatter plot of all datasets. The horizontal coordinate is the average of the red-green opposite color intensity channel of each image, and the vertical coordinate is the average of the yellow-blue opposite-color intensity channel. Images were converted from the RGB color space to the Lab color space. The ‘a’ channel represents red-green opposite color intensity; if the positive value is higher, then it is redder, otherwise it is greener. The ‘b’ channel represents the strength of yellow-blue opposite colors; if the positive value is higher, then it is more yellow, otherwise it is bluer. The horizontal coordinate is the average ‘a’ channel of each image, and the vertical coordinate is the average ‘b’ channel.

Detailed descriptions of the MSHF dataset is shown in Table 2. To show the diversity of the images in the MSHF dataset, we converted all images from the RGB color space to the Lab color space, and created a spatial scatter plot to show the distribution, as shown in Fig. 2. There are seven kinds of symbols in the figure, representing the seven subsets.

**Table 2:** Basic information on the multi-source heterogeneous fundus (MSHF) dataset.

Image type	Device	Name	Number	FOV	Resolution	Disease
CFP images	Kowa Nonmyd fundus camera	DR-XJU	235	45 degrees	1924 × 1556	DR
	TRC-NW8 fundus camera	DR-ZJU	187	50 degrees	1924 × 1556	DR
		Glaucoma	52	50 degrees	1924 × 1556	Glaucoma
		Healthy	26	50 degrees	1924 × 1556	/
Portable camera images	DEC200 portable fundus camera	Local1	199	60 degrees	2560 × 1960	/
		Local2	103	60 degrees	2560 × 1960	/
UWF images	Optos ultra-wide field imaging system	UWF-mosaic	500	200 degrees	1924 × 1556	DR

### Quality evaluation

To facilitate the clinical application, the evaluation standard is a generic quality gradation scale that adhered to the generic-but-not-structural principle, as listed in Table 3. The overall quality represents the general impression of the images, and suggests whether or not the image is useable, while the illumination, clarity and contrast are parameters based on the characteristics of human visual system, and indicates the potential aspects to improve the image quality.

**Table 3:** Generic quality gradation scale.

Item	Principle description
Illumination	0: The image is overexposure, underexposure, or with the uneven illumination or color.
	1: The image has good illumination and color.
Clarity	0: The image has noticeable blur in optic disks, vessels, or background.
	1: The image is clear, with no blur in the field.
Contrast	0: Image contrast is low such that the band of pixel intensity is narrow, making it difficult to distinguish the vessel or lesions.
	1: Image has good contrast.
Overall quality	0: The image quality is bad, making it hard to diagnosing.
	1: The image quality is good, and can be used for diagnosing.

**Fig. 3:**
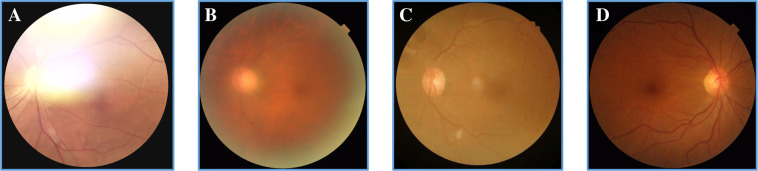
Examples of images of low and high quality. (**A**–**D**) are CFP images, (**E**–**H**) are portable camera images, and (**I**–**L**) are UWF images. (**A**), (**E**) and (**I**) show the presence of uneven illumination or color. (**B**), (**F**) and (**J**) show the presence of blur that affect the clarity. (**C**), (**G**) and (**K**) show the presence of low contrast. (**D**), (**H**), and (**L**) are images of good quality in every aspect.

Images of the MSHF dataset were labelled by three ophthalmologists according to the principle. If the image was of good quality in a particular category, it was marked as ‘1’, and if not, as ‘0’. The ground truth was decided by the majority rule. Examples of high- and low-quality images of CFP, portable camera and UWF are shown in Fig. 3.

### Dataset division

To make the MSHF dataset applicable for further AI model building, the dataset was manually divided into the training set (80%) and the test set (20%). The training set was used for learning and the test set was used for testing. There was no intersection between the 2 sets, and the variety of images was distributed equally. The 2 sets contained basically equal ratio of good- or poor-quality images. It is worth noting that we offered a possible way of split, and we did not mean to restrict its use. Future researchers can freely use this data set to achieve their research purposes.

## Data Records

The MSHF dataset has been uploaded to Figshare in the form of a zipped file^17^. The unzipped file folder contains the original fundus photographs and quality evaluation scores. The unzipped file is organized into 2 folders and 2 Microsoft office Excel list, named “Original”, “AI-use”, “MSHF_quality_scores.xlsx” and “Individual_scores.xlsx”, respectively. The “Original” folder contains 3 subfolders, named “CFP”, “Portable_camera” and “UWF-mosaic”. Among them, the “CFP” folder is consisted of “DR-XJU”, “DR-ZJU”, “Glaucoma” and “Healthy”, and the “Portable_camera” folder is consisted of “Local1” and “Local2”. Images in these 7 folders are stored, named and arranged in the same way. The “AI-use” folder contains “train” and “test” subfolders consisting of 1042 images recommended for training and 260 images for testing. Images are named the same as they are in the “Original” folder. The current data split strategy is proposed by our team and might be subject to change for other research purposes. In the file “MSHF_quality_scores.xlsx”, there are 7 sheets corresponding to the 7 subfolders “DR-XJU”, “DR-ZJU”, “Glaucoma”, “Healthy”, “Local1”, “Local2” and “UWF-mosaic”. Five columns are presented in each sheet. The first column represents the name of each image. The subsequent columns indicate the final score of “illumination”, “clarity”, “contrast” and “overall quality” of the image. In the file “Individual_scores.xlsx”, there are scores of each image annotated by three annotators. The score of each item is either 1 or 0.

## Technical Validation

### Dataset characteristics

There are 1302 fundus images and their corresponding quality labels in the MSHF dataset. These images are acquired from 952 subjects. The mean age of the subjects was 51 years, with a standard deviation of 20.12 years. There were 602 images from female subjects and 700 from males. All the subjects were Asian. Detailed annotation of the dataset is presented in Table 4.

**Table 4:** Dataset annotations.

Item	Illumination		Clarity		Contrast		Overall quality	
Name	0	1	0	1	0	1	0	1
DR-XJU	36	199	120	115	78	157	117	118
DR-ZJU	31	156	34	153	6	181	40	147
Glaucoma	45	7	48	4	42	10	50	2
Healthy	2	24	0	26	0	26	0	26
Local1	158	41	94	105	85	114	142	57
Local2	78	25	59	44	41	62	77	26
UWF-mosaic	215	285	163	337	50	450	168	332

Images from healthy volunteer are generally in good quality, in terms of every aspect. Images from DR patients have mixed results of overall quality, and good-quality images account for about 60%. However, nearly all images from glaucoma patients are in bad quality. It might be explained that glaucoma patients are generally the elderly, and their pupil cannot be dilated because of the intraocular pressure. The distribution of poor-quality images increases the diversity of the MSHF dataset, as shown in Fig. 2. There are clear differences in the color distributions of the different sub-datasets, and UWF-mosaic differs significantly from other datasets. Glaucoma dataset is also special, because the distribution seems random. LOCAL_1 and LOCAL_2 almost overlap each other, and DR-XJU is a little different from DR-ZJU. The characteristics of the MSHF dataset make it similar to clinical scenarios, and in AI area, the diversity can help to develop robust algorithms.

### Inter-annotator consistency

To evaluate the inter-annotator consistency of our dataset, Fleiss Kappa coefficients between the annotators of the four aspects were calculated. The Fleiss Kappa coefficient of ‘contrast’ was 0.786, indicating a substantial agreement, and the results of ‘illumination’, ‘clarity’ and ‘overall quality’ was 0.820, 0.804 and 0.848, suggesting an almost perfect agreement.

## Usage Notes

The entire dataset can be downloaded from the link mentioned above. It should be mentioned that the data split strategy was made considering the quality score and the variety of data source, and we did this for the convenience of further artificial intelligence use. For researchers who use the dataset for other purpose, we expect them to cite this paper in their research output and acknowledge the contribution of this dataset in their study.

## Data Availability

No novel code was used in the construction of MSHF dataset.
